# Supplementary material for: The effect of framing on attitudes towards Alzheimer’s disease. A comparative study between younger and older adults
Source: PLoS One. 2022 Jul 7;17(7):e0270959. doi: 10.1371/journal.pone.0270959 (PMC9262181; doi:10.1371/journal.pone.0270959)
Supplement: Attachment — Submitted filename: Response to Reviewers.docx [file pone.0270959.s001.docx]

May, 2022

Perla Werner

Academic Editor of *PLOS ONE*

Dear Academic Editor,

Firstly, I would like to thank you for reviewing our work and giving us the opportunity to review our paper “*What does Alzheimer’s disease mean for younger and older people? An analysis of framing influence in emotions and attitudes towards Alzheimer-type dementia*”.

**Manuscript ID:** PONE-D-22-09110

We have written a point by point cover letter indicating the changes we have made in the manuscript and the responses to each of journal requirements and the comments of the reviewers.

**Journal requirements:**

1. We have ensured that our manuscript meets PLOS ONE’s style requirements (e.g. figures and tablets format).
2. With respect to Figure 2, all the images used were downloaded from the *Pixabay* website and were free of copyright under the Creative Commons CC0 license, so these images can be used and published freely. We have provided this information in Fig 2 legend.
3. We have ensured that our title page meets all requirements.
4. We have included citations 42 and 48, which have been included in the reference list. We have marked them in red so you can see it.

**Response to reviewer 1:**

1. As suggested, we have modified the title of manuscript (before: *What does Alzheimer’s disease mean for younger and older people? An approach to the effect of framing*; now: *The effect of framing on attitudes towards Alzheimer’s disease. A comparative study between younger and older people*).
2. With regard to your comment about the frames interpretation, we have explained more on the application of a frame may lead a particular interpretation. **(Page 5-6, lines 89-95).**
3. As suggested, we have added the question asking impact and emotions experienced by the participants. **(Page 10, lines 225-226).**
4. The data was not collected during the pandemic, so we cannot discuss the Covid-related impacts on methodology, since there was no restriction or rule related to the pandemic that prevented the normal development of data collection.
5. We agree with you. Older participants in this study are the young-old, so we have added the oldest-old like an interesting sample that includes in future studies. **(Page 23, line 491).**
6. As suggested, we have discussed the implications of age differences in relation to choosing messages framed to reduce stigma. **(Page 23-24, lines 472-479).**

**Response to reviewer 2:**

*Conceptual and stylistic issues:*

1. Regarding your comment, we have been more thorough with the characterization of Alzheimer’s disease. For example, we have explained that Alzheimer’s behavioural and psychological disorders (neuropsychiatric symptoms) have been associated with stigma towards AD **(Page 4, lines 44-47).**
2. We have softened the sentence as you suggested **(Page 4, lines 53-54).**
3. Regarding your doubt about if we men “an audience” instead of “various audiences”, we have specified what we mean which “various audiences” (**Page 5, lines 81-82).**
4. We understand the incongruence that you indicate about happiness, but we need answer about all basic emotions (happiness, sadness, fear, anger, and disgust). In this context (chronic neurodegenerative disease), we know that people don’t answer with high levels of happiness, and we don’t intend it. On the other hand, some body-mind unity frame’s affirmations (e.g. she will lose some of her abilities, but she will still feel emotions) can provoke feeling of consolation or tenderness; in short, emotions congruent with happiness. In this way, we have explicated it. Moreover in “Results” section, we have indicated that both groups experienced low-medium levels of happiness **(Page 19, lines 368-369).**
5. We have included the information provided by you about Stites et al. (2018). **(Page 6, lines 112-115).**
6. We have specified more about the effect size of 0.25 and we have referenced it. **(Page 10, lines 192-193 and lines 195-196).**
7. As suggested, we have included an example of questions about level of impact the campaign had on participants. **(Page 10, lines 225-226).**
8. With regard your comment about “positivity” and “positive messages”, we have clarified what we mean and eliminated confusing concepts. **(Throughout the “Discussion” section, e.g. page 21, lines 423-425).**

*Minor comments:*

1. With regard your comments about abstract: (1) we have corrected the abstract’s error (‘these’ rather ‘this’) **(page 2, line 34)**, (2) we have added the statistical test (**Page 2, line 26 and 28-29)**, and (3) we have reported more information about frames examined for the two age groups **(Page 2, lines 22-25).**
2. Age ranges are added. We have underlined them in yellow so you can see them. **(Page 9, lines 186 and 187).**
3. Regarding your comment about cell sizes in the tables, we have included sample size.
4. With respect your comment about illustrate with examples, we have clarified why we knew that the participant had positive attitudes towards disease. **(Page 22, lines 431).**
5. With regard your question about how the groups were randomly divided, we have explained how the randomization was carried out **(Page 11, lines 252-253).**

We hope that these changes have improved the manuscript and you will consider it for publication in *PLOS ONE*.

Yours sincerely,
